# New Structures and Gating of Voltage-Dependent Potassium (Kv) Channels and Their Relatives: A Multi-Domain and Dynamic Question

**DOI:** 10.3390/ijms20020248

**Published:** 2019-01-10

**Authors:** Francisco Barros, Luis A. Pardo, Pedro Domínguez, Luisa Maria Sierra, Pilar de la Peña

**Affiliations:** 1 Departamento de Bioquímica y Biología Molecular, Universidad de Oviedo, Edificio Santiago Gascón, Campus de El Cristo, 33006 Oviedo, Asturias, Spain; pdluengo@uniovi.es (P.D.); pdelapena@uniovi.es (P.d.l.P.); 2 Oncophysiology Group, Max Planck Institute of Experimental Medicine, Hermann-Rein-Strasse 3, 37075 Göttingen, Germany; pardo@em.mpg.de; 3 Departamento de Biología Funcional (Area de Genética), Instituto Universitario de Oncología del Principado de Asturias (IUOPA), Universidad de Oviedo, 33006 Oviedo, Asturias, Spain; lmsierra@uniovi.es

**Keywords:** ion channels, Kv channels, structure, molecular architecture, gating, electro-allosteric coupling

## Abstract

Voltage-dependent potassium channels (Kv channels) are crucial regulators of cell excitability that participate in a range of physiological and pathophysiological processes. These channels are molecular machines that display a mechanism (known as gating) for opening and closing a gate located in a pore domain (PD). In Kv channels, this mechanism is triggered and controlled by changes in the magnitude of the transmembrane voltage sensed by a voltage-sensing domain (VSD). In this review, we consider several aspects of the VSD–PD coupling in Kv channels, and in some relatives, that share a common general structure characterized by a single square-shaped ion conduction pore in the center, surrounded by four VSDs located at the periphery. We compile some recent advances in the knowledge of their architecture, based in cryo-electron microscopy (cryo-EM) data for high-resolution determination of their structure, plus some new functional data obtained with channel variants in which the covalent continuity between the VSD and PD modules has been interrupted. These advances and new data bring about some reconsiderations about the use of exclusively a classical electromechanical lever model of VSD–PD coupling by some Kv channels, and open a view of the Kv-type channels as allosteric machines in which gating may be dynamically influenced by some long-range interactional/allosteric mechanisms.

## 1. Introduction

Ion channels are macromolecular (mostly gated) pores that permit the passive flow of ions across the membrane down their electrochemical gradients. Unlike other ion transport systems, they also show high unitary transport rates, typically orders of magnitude higher than any other transporter type. Although some ion channels can operate as permanently open entities, their opening and closing is usually controlled by a variety of chemical and/or physical (e.g., mechanical, thermic, and electrical) stimuli, leading to two basic channel types according to their mode of operation: ligand-gated and voltage-gated ion channels. Voltage-gated channels, such as those belonging to the voltage-dependent K^+^ (Kv) subfamily that represent the central issue on this report, constitute molecular machines that include a mechanism for opening and closing (gating) triggered by a change in the magnitude of the transmembrane voltage. The concept of protein conformational modifications driven by the change in energy that occurs upon binding of a ligand is well established: part of the energy liberated by binding to a high affinity site may be used to drive an inherently unfavorable conformational transition [1]. On the other hand, all transmembranal proteins have evolved to survive and function in an intense electric field which, at a resting membrane voltage of 100 mV across a membrane with a thickness of 100 Å, corresponds to 100 kV/cm, close to the dielectric breakdown point where electrostatic force rips molecules apart, and about an order of magnitude larger than the 14 kV/cm triggering a lightning discharge in air during a thunder storm [1,2]. Therefore, some membrane proteins (e.g., the voltage-dependent Kv channels) have also evolved to use the electric field power for moving some of their charged groups crosswise through the membrane, leading to shape changes and to opening and closing transitions when a transmembrane voltage variation occurs [1].

It is important to note that voltage-dependent gating (even when only referring to activation/deactivation mechanisms) involves at least three molecular processes: (i) detection of transmembranal voltage gradient changes by a voltage sensor domain (VSD) and its subsequent conformational rearrangement(s), known as VSD activation; (ii) propagation of the VSD movements to the pore-gate domain (PD) that mediates ion permeation, also known as VSD–PD coupling; and (iii) opening of the PD gate, leading to pore opening and ion conduction [3]. In this review, we will analyze several aspects of the VSD–PD coupling, according to recent advances in the knowledge of channel architecture in several members of the six-transmembrane domain one-pore domain subfamily (6TM1P) to which the Kv channels belong, and to some new functional data obtained with channel variants in which the covalent continuity between the VSD and PD modules has been interrupted. These advances and new data bring about some necessary reconsideration regarding the sufficiency of a classical electromechanical lever-based mechanism alone to explain VSD–PD coupling in some Kv channels, opening a view of the Kv-type channels as allosteric machines, in the sense that gating may be dynamically influenced by conformational changes that can be distant from the actual gate. Note also that the voltage-dependent response of many of these entities can be extended to the inactivation gating, in which following the initial opening of the activation pore gate, secondary gating transition(s) take place as a consequence of a maintained change in voltage, leading to a delayed non-conducting conformation (reviewed in [4]). Nevertheless, only mechanisms of primary channel opening and closing (activation and deactivation gating) will be considered here.

## 2. Cryo-EM: A New Catalog of Kv and Other Ion Channel Structures

Kv channels belong to a group of ion channels also known as “S4” or “6TM1P” subfamily of the pore-loop family [5,6,7,8]. They are composed by a four transmembrane helices (TMs) domain, which has been added to the N-terminal end of every single subunit of a typical tetrameric pore-loop protein that constitutes the ion-permeation pore moiety [5,7]. Thus, Kv channels are tetramers in which each subunit contains six TM segments, S1 to S6. The assembled tetramer of a functionally complete Kv channel shows a modular organization with a pore domain (PD) in which the S5–S6 sections of the four subunits together are arranged to leave a square-shaped ion conduction pore at the center, that is surrounded by the four voltage-sensing domains (VSDs) located at the periphery. Every VSD is formed by TMs S1 to S4; the primary voltage sensitive component is helix S4, containing the positively charged residues that move in response to changes in membrane potential. This general architecture of the Kv channels is also shared by other S4-pore-loop/6TM1P channels (not all of them activated by voltage) with some subtle variations, including: (a) the addition of a fifth TM helix to the beginning of the VSD module in the large-conductance Ca^2+^-activated K^+^ (BK) channel; (b) the almost total absence of positively charged residues in the S4 of some members of the family (e.g., some transient receptor potential or TRP channels); (c) the fusion of the four monomers in a single polypeptide in the voltage-gated Ca^2+^ and Na^+^ channels, where four similar repeats with six TMs mimic the tetrameric assembly of the Kv channels [4,5]. Finally, the concept of Kv channels being the result of an evolutive combination of two functionally autonomous VSD and PD modules [9,10,11] is reinforced by: (i) the existence of PD-only voltage-independent channels [12,13,14,15] that probably share a common ancestor with Kv and other voltage-dependent channels [11,16,17]; (ii) the demonstration of functional VSD-only-based voltage-dependent channels [18,19,20] and of voltage-controlled enzymes in which a VSD resembling those found in voltage-gated channels provides membrane potential control of the catalytic activity [21]; and (iii) the generation of voltage-gated ion channels by either fusing together VSDs and PDs from different sources [22,23,24], or co-expressing them as separate protein entities [25,26,27,28].

Until very recently, the good knowledge of the common general topology of the S4-pore-loop/6TM1P channels has not been accompanied by a detailed knowledge of their structural characteristics and/or differences. Despite the relevance of several methods for high-resolution structure determination (e.g., X-ray crystallography or NMR spectroscopy) to get a better knowledge of the tridimensional organization of Kv and other ion channels, the catalogue of solved channel structures remained modest, since this type of macromolecules were difficult to crystallize or too large to study with NMR. Indeed, although the tridimensional structure of the transmembrane core of some Kv channels was solved at high resolution [29,30,31], the complete architecture of an almost full-length Kv channel remained as a challenge, particularly concerning the cytoplasmic channel domains. These domains are in many cases considerably larger than the transmembrane core region, and show far more divergence than the rest of the molecule does, even among closely related channels. Furthermore, some interactions between them and the transmembrane part of the channel can critically influence the gating properties and other channel functionalities, either directly or in response to exogenous modulators [4]. However, the use of high-resolution structure determination methods in a “divide and conquer” approach [32] has provided partial structural information to be integrated with biochemical, electrophysiological and spectroscopic data, yielding a better picture of channel organization on a domain-by-domain basis [33]. This has been used to gain new insights into the overall channel architecture, although it has been difficult to assign the solved structures to specific functional protein states and to ascertain whether they accurately represent the conformation of the full protein in its native membrane environment. 

Over the last few years, cryo-electron microscopy or single-particle cryo-EM (cryo-EM) has been used to yield a panoply of high-resolution structures of ion channels, including several Kv and other S4-pore-loop/6TM1P channels (see below). The advantages of this method to solve structures of this type of macromolecules include: (i) the possibility to study them in more ‘native’ biochemically functional conditions, as opposed to the specific and often technically difficult conditions in which the membrane molecules may happen to crystallize; (ii) the possibility to determine structures in conformations that are not necessarily those selected for crystal packing forces. These alternative conformations may correspond to more functional states, while some times the rigid structures needed for X-ray crystallography would represent a most stable state, not necessarily the most common or representative state in the cell; (iii) the little volumes and lower concentrations of protein sample necessary to obtain the cryo-EM data, that can be more easily obtained by direct purification from natural sources; and (iv) the possibility to avoid extensive protein engineering to remove flexible regions and/or addition of stabilizing substrates that may be required to make the molecule crystallize. Although in this case the cryo-EM data may lack resolution in the flexible/peripheral regions, they can provide some insight into the natural dynamics of the molecule by defining the small disordered domains present in a macromolecule or attached to the ordered core of a larger complex. Complementary crystallographic definition of these flexible structures stabilized in a crystal lattice can be used to define their ordered architecture and subsequently dock them into the low-resolution regions obtained by cryo-EM. Indeed, the complementarity of cryo-EM and other more conventional methods of structure determination (such as X-ray crystallography and NMR spectroscopy) has been also highlighted [34,35,36]. More specific details about the cryo-EM methodology and its possible limitations and drawbacks have been also covered in some excellent recent reviews [37,38,39,40,41,42,43] and will not be further considered here.

## 3. Swapped or Non-Swapped Organization of the Kv Structures?

**Figure 1:**
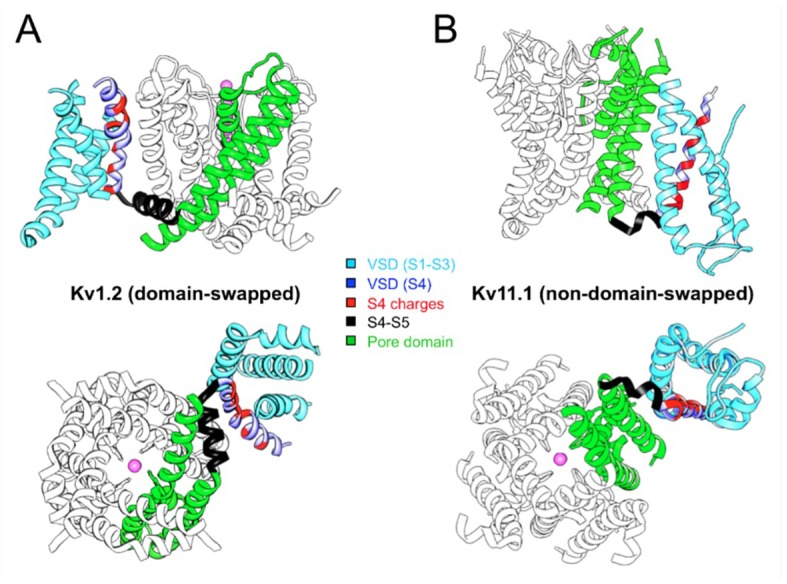
Comparison of domain-swapped (**A**) and non-domain-swapped (**B**) architectures of Kv channel transmembrane domains. Only a single VSD is shown attached to the tetramer of the PD for clarity. One complete subunit is shown colored as indicated. The magenta balls indicate the position of K^+^ ions in the selectivity filter. Note the different length of the S4–S5 linker determining the VSD contact with the neighboring (**A**) or the same (**B**) subunit. Structures viewed from the membrane plane (top) and from the cytoplasmic side (bottom) are shown for Kv1.2 (PDB: 2A79) and Kv11.1 (hERG, PDB: 5VA2).

**Figure 2:**
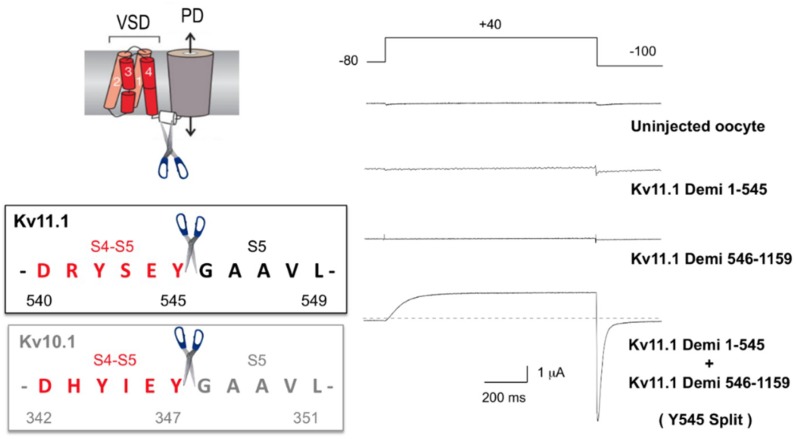
Physical continuity between the voltage-sensing and the pore modules is not necessary for KCNH (Kv11.1-hERG and Kv10.1-eag1) channel functional expression. A scheme of the transmembranal core of a channel subunit is shown on the upper left corner. The aminoacid sequence of the S4-S5 linker region and the point at which the covalent link between the VSD and the PD is interrupted to generate S4-S5 split channels is shown on the lower left part. Electrophysiological recordings of Kv11.1 (hERG) N- and C-terminal demi-channels injected alone or together in *Xenopus* oocytes bathed in 50 mM high-K^+^ medium and submitted to the voltage-clamp protocol indicated on top of the traces are shown on the right. Note the presence of typical hERG outward current traces during the depolarizing step and the prominent inward tail upon repolarization, only when the amino and carboxy channel halves are combined. Modified from [25].

The recent structural data of several Kv channels allowed to ascertain that they adopt two main general organizations in their transmembrane domains (Figure 1): (i) Those in which the peripheral VSDs contact the PD of a neighboring subunit, leading to the domain-swapped architecture typical of the Kv1 to Kv9 families (*Shaker*-type [29,30,31,44,45]). This architecture is also shared by Nav and Cav voltage-gated sodium and calcium channels [46,47,48,49], and by the Kv channel relatives, generally non-selective cation channels from the transient receptor (TRP) family (reviewed in [50]). (ii) Those in which the VSDs contact the PD from the same polypeptide, determining a non-domain-swapped architecture. This last feature has been demonstrated for members of the Kv10 and Kv11 families [51,52], but it is also present in the Slo1 and SK Ca^2+^-activated K^+^ channels [53,54,55], the Na^+^-dependent K^+^ channel Slo2.2 [56], the HCN1 hyperpolarization-activated cation channel [57] and the prokaryotic and eukaryotic cyclic-nucleotide-gated channels [58,59]. These two structural alternatives have been associated to the fact that in the domain-swapped channels a long α-helical S4-S5 linker connects the VSD and PD domains and cuffs around the helical bundle formed by the S6 helices that constitutes the cytoplasmic channel gate [60,61,62,63,64,65,66,67,68,69,70], but see also [71]. In this case, the linker would function as a mechanical lever (electromechanical lever coupling) that transmits the force generated by the VSD conformational reorganizations to the gate in order to control activation and/or deactivation channel gating [44,72,73,74,75,76,77]. In contrast, in the non-domain-swapped architectures a short and non α-helical S4–S5 linker is mostly found, pointing to the existence of an alternative mechanism to control channel gating, even in those Kv channels that operate in a voltage-dependent way but share this molecular organization. Indeed, pioneer studies with Kv10.1 and Kv11.1 channels demonstrated that breaking the covalent link between the VSD and the PD at the level of the S4–S5 linker (S4–S5 split channels), allowed the formation of voltage-gated channels showing an almost unaltered voltage-dependent gating, that recapitulates both the voltage-sensing and permeation properties of the complete protein [25] (Figure 2). Therefore, at least in these Kv channels, the length of the S4–S5 linker would not allow voltage-dependent channel activation through the linker acting as a rigid mechanical lever which, following the outward movement of the S4 voltage-sensor helix, pulls apart the N-terminal portion of S5 and the C-terminal end of S6 to open the cytoplasmic channel gate [44,72,73,74,75,76,77]. Thus, these structural and functional evidences have been recently regarded as a solid indication that in those voltage-dependent channels lacking a long and alpha-helical S4–S5 linker (e.g., Kv10, Kv11, Slo1 and HCN channels), a different mechanism should explain coupling voltage sensing to pore gating. In this new mechanism, the non-swapped voltage sensors should work to transmit force and regulate the gate in a way somewhat different than trough the lever mechanism proposed for *Shaker*-like Kv channels [28,51,52,54,57,78,79].

It is important to note that although the length of the S4–S5 linker can act as an essential factor for domain swapping, other regions may also determine the structural organization of the channel. Thus, a non-swapped architecture has been recognized in small conductance Ca^2+^-activated K^+^ channels that contain a quite long S4–S5 linker with two α-helices, instead of the short turn encountered in other non-domain-swapped channels [55]. Conversely, a domain-swapped organization has been observed in TRPM8 channels, in which no obvious or only a short S4-S5 linker exists, following a longer and straight C-terminal part of the S4 helix [80]. Finally, it has been reported that a single point mutation in the S5 transmembrane helix of the TRPV6 channel converted the domain swapped architecture of the channel to non-domain-swapped, even though the full–length of the protein was preserved [81]. Interestingly, in contrast to the lack of domain swapping in the transmembranal core of Kv11.1, there is extensive domain swapping between subunits within the cytoplasmic regions of the channel [52]. This opens the possibility that the gating functionality is not only related to the swapped organization of the channel core, and that the swapped architecture of the cytoplasmic domains constitutes a crucial component of the gating machinery, or an important regulator of the gating system. This means that the functional output may depend on the structural organization of the protein, including the molecular architecture of the transmembrane core, but also that Kv channels and their relatives operate as allosteric machines in which the final function (e.g., the gating machinery operation) can be strongly influenced by other, sometimes very distant, components of the ensemble. To support this view, the existence of important cytoplasmic domain influences in the gating mechanisms of the typical domain-swapped Kv channels and their relatives, and evidence about allosteric coupling acting on them is considered next.

## 4. Allosteric Influences in the Coupling Mechanisms of Domain-Swapped Kv Channels and Their Relatives

Although the existence of a ‘rigid lever’ electromechanical coupling mechanism is a feature of the Kv and some other 6TM1P related channels that present a domain-swapped architecture, there are evidences that allosteric mechanisms can be present in some of them either influencing gating and/or representing essential constituents of the gating machinery. 

Among those, transient receptor potential channels (TRPs) constitute a family of generally non-selective cation channels involved in sensory transduction processes, both in unicellular and multicellular organisms, either at the level of the entire organism or at the single cell level [82,83]. TRP channels have been grouped in seven subfamilies [84,85], from which almost 50 cryo-EM structures have been resolved at different resolution (reviewed in [50]). Interestingly, all TRPs share a common architecture analogous to that of Kv channels, comprising: (i) a homotetrameric assembly (although heterotetramers have been reported; reviewed in [86]) with the permeation pathway at the four-fold symmetry axis; (ii) a pore region formed by the transmembrane segments S5 and S6, carrying also an intervening re-entrant pore helix and pore loop between them, and four voltage sensor-like domains surrounding the pore, each with four transmembrane S1–S4 helices. Furthermore, so far all of them adopt the domain-swapped organization common to many voltage-gated ion channels [84]. Additionally, TRP channels contain extended cytoplasmic N-terminal and C-terminal domains involved in the regulation of function and trafficking. These domains widely differ when comparing different TRP channels, with very diverse organization of protein motifs, such as ankyrin repeat, PDZ, or kinase domains [82]. Noticeably, despite the presence of VSD-like domains, few TRP channels show voltage-dependence of gating or a clear voltage-dependent activation [80,82,87,88,89,90,91]. This is probably due to the amino acid sequence in the fourth transmembrane helix, which for the voltage-gated TRPs includes some positively charged residues and for the voltage-independent ones does not. Instead, TRPs are polymodal channels that transduce multiple stimuli of different nature into allosteric changes [83,92,93]. The stimuli may be physical (voltage, temperature, pressure), the binding of chemicals (agonists, lipids, ions), or direct interaction with other proteins. Furthermore, the weak voltage dependence of several TRP channels, extending into non-physiological positive voltage ranges, can be strongly shifted towards physiologically relevant potentials by stimuli such as temperature or ligand binding. This shift is favored by the small gating charge of the TRP channels, and constitutes an important factor for their gating versatility [94].

Despite the structural divergence in the cytoplasmic regions of the different TRPs, two features relevant to gating are shared for most of them: a quite long S4–S5 linker connecting the VSD-like domain and the S5-pore-S6 segment, and a TRP domain (also called TRP box or TRP helix [95]) constituted by an amphipathic helix following S6. The TRP domain forms extensive interactions with the S4–S5 linker (and the pre-S1 region), what has been considered as a crucial factor for the allosteric coupling to gating in response to a variety of stimuli [96]. Thus, the S1–S4 and TRP domains form a tightly associated entity directly coupled to the intracellular gate of the pore, with S1–S4 connected to S5 through the S4–S5 linker, and the TRP helix directly linked to the pore-lining S6 helix. As lucidly discussed in [83], this is reminiscent of the well characterized allosteric communication among ion-channel sensing modules in the calcium- and voltage-sensitive BK channel (see below, non-domain-swapped section). Therefore, the S4–S5 linker/TRP domain assemblage has been repeatedly viewed as a central component of the S1–S4/TRP gating apparatus. Indeed, mutations in the TRP box and the S4–S5 linker similarly affect the voltage and ligand activation of TRPM8 [97], consistent with the proposed role for this domain(s) as the coupling machinery transferring the chemical energy of ligand binding to pore opening [83]. The TRP domain has also been recognized as a key structural determinant for interaction with PI(4,5)P_2_, a well-known gating modulator for most members of the TRP channel superfamily [83]. Finally, based in the fact that a number of pathogenic mutations within the S4–S5 linker of several TRPs constitute gain-of-function factors leading to constitutively active channels, a role for this linker as the gearbox for TRP channel gating has been highlighted (reviewed in [95]). In summary, apparently different cytoplasmic regions, including some at the N- and C-terminus, contribute to the TRP channels coupling system that communicates their several sensors with the pore gate.

Aside from the Kv-related TRP channels, it has been proposed that some Kv channels with a domain-swapped organization (i.e., included in the Kv1 to Kv9 group), can use a coupling mechanism based in a ligand/receptor model, in which neither ‘attractive’ nor ‘repulsive’ force would be necessary to be exerted by S4 on the gate [98,99]. This model would be different from the ‘attractive’ or ‘repulsive’ electromechanical types of coupling in which the S4 voltage sensors pull or push the gate, respectively, to open or constrict it via the S4-S5 linker and S6 interaction [74,100]. This is the case of the Kv7 (KCNQ) channels, a group with great physiological relevance in the kidney, gastro-intestinal tract, brain, and heart, that when mutated, can lead to cardiac, neurological, and other disorders (reviewed in [7,101,102]). Thus, using soluble peptides corresponding to the S4–S5 linker or to the carboxy end of the S6 helix of Kv7.1 (KCNQ1/KvLQT1), it has been demonstrated that the S4–S5 linker peptides (acting as ‘ligand’ or inhibitory peptides) reduce the voltage-dependent channel current. Alternatively, the end-of-S6 peptides (acting as ‘receptor’ or decoy peptides) increase channel activity, by competing with the endogenous end-of-S6 sequences that bind the inhibitory S4-S5 linker regions [98]. These findings led to conclude that these channels use a more labile ligand/receptor mechanism of gating, in which the S4-S5 linker could act as a ligand that binds to the base of helix S6, locking the channels in a closed conformation, instead of using the canonical electromechanical lever coupling mechanism used by some *Shaker*-like channels [98,99]. The interaction between S4–S5 and S6 is counteracted upon depolarization, when the S4–S5 linker is dragged away from its receptor site near S6, allowing for a dilation of the S6 helices bundle that constitutes the cytoplasmic gate [99]. The existence of an allosteric coupling mechanism between the voltage sensor and the pore domains for voltage-dependent gating of KCNQ1 [3,73], but also of M-channels formed by KCNQ2 and KCNQ3 heteromers [103], has been also emphasized. In fact, an allosteric gating model for these channels has been viewed as a clue to explain their regulation by PIP_2_, that acts as an essential requirement for proper VSD–PD coupling [45] and subsequent voltage-dependent gating of the KCNQ family channels [3,99,103].

It is important to note that even in Kv channels with prototypical domain-swapped organization, and a S4–S5 linker lever-type of electromechanical VSD–PD coupling for gating, the covalent connection of both modules via S4–S5 would directly track only the S5 helix. Therefore, to gate these channels some additional non-covalent interactions will be necessary to track the helices bundle that forms the pore gate at the bottom of S6 [60,61,62,63,64,65,66,67,68,69,70]. Thus, apart from the central role of the S4–S5 linker for electromechanical coupling, an intersubunit interaction between the lower S4 and the S5 of the neighboring subunit seems to be essential for the final cooperative gating transition that leads to pore opening in *Shaker*-like Kv channels [104,105,106]. Indeed, a non-canonical coupling pathway, based in specific interactions between residues from S4 and S5, has been also shown to be involved in voltage-dependent activation gating of the prototypical *Drosophila Shaker* channel [107]. Finally, important influences of the Kv1.1 and Kv1.2 N-terminal cytoplasmic T1 domain on gating have been demonstrated. This supported the conclusion that the polar T1 subunit interfaces play a key role in the conformational changes that lead to opening in these channels, making the authors propose that these channels are big, allosteric machines, in which the T1 domain could act like the cytoplasmic domains in calcium-sensitive potassium channels and cyclic-nucleotide-gated and hyperpolarization-activated cation channels [108,109] (reviewed in [4,110]). In what proportion those domains and interactions constitute a bona fide component of the coupling machinery, or simply indirectly influence it, remains to be established. Also, the possibility that other cytoplasmic structures/domains are part of the coupling process, or act as allosteric modulators, remains as an open question. Interestingly, although perhaps necessary, maintenance of the S4–S5 covalent continuity is not enough for effective gating of Kv7.1 channels, since they also need PIP_2_ interactions with the initial S4–S5 linker region for functional VSD–PD coupling and voltage-dependent channel opening [6,45,103].

## 5. Allosteric/Interactional Modulation of Coupling in KCNH Family and Other Non-Domain-Swapped Kv-Like Channels

As indicated above, recent elucidation of a number of ion channel molecular structures, demonstrated that several Kv channels (e.g., Kv10 and Kv11), and also other members of the S4-pore-loop/6TM1P subfamily gated or not by voltage (e.g., Slo1, Slo2.2, SK, HCN1, and CNG channels), show a non-domain-swapped architecture [51,52,53,54,55,56,57,58,59]. It has been repeatedly indicated that this molecular organization is hardly compatible with the concept of a canonical electromechanical coupling, based in a rigid S4–S5 linker mechanical lever as main coupling mechanism [28,51,52,54,57,78,79]. Furthermore, evidences for involvement of allosteric/interactional steps leading to gating in some of these entities, have been reported and are considered next.

### 5.1. Non-Domain-Swapped Kv Channel Relatives

SK: Small-conductance Ca^2+^-activated K^+^ (SK) channels belong to the SK/IK group of the Ca^2+^-activated K^+^ channels (KCa) family [111], that also includes the BK (big conductance, big potassium, maxiK, KCa1.1, *KCNMA1* or Slo1) channel (see below). BK or Slo1 is also part of the *Slo* gene channel subfamily, in which other Na^+^-activated and H^+^-inhibited K^+^ channels are included [111,112,113]. Even though all these channels belong to the 6TM1P class of ion channels with VSD and a PD, Ca^2+^-dependent gating of SK channels is exclusively performed through a ligand-mediated mechanism that uses a Ca^2+^-bound calmodulin (Ca^2+^-CaM), associated to a cytoplasmic CaM binding domain (CaMBD) of the channel, to open the permeation pathway with very little or no voltage dependence [113,114]. Therefore, SK channels depend on an allosteric mechanism to communicate the Ca-CaM binding to opening the channel. The recent cryo-EM elucidation of the structure of a human SK4-CaM channel complex, both in the Ca^2+^-free closed and the Ca^2+^-bound open conformations [55], has provided new insights into the molecular mechanisms involved in SK channel gating. Interestingly, SK4 exhibits a non-domain-swapped architecture, even though it contains a long S4–S5 linker, constituted by two α-helices (residues 177–186 and 192–202) and a short turn, in contrast with the very short S4–S5 linker present in other non-domain-swapped 6TM1P channels. It has been proposed that, in this case, the linker structure may be suited to confer the CaM-mediated Ca^2+^ sensitivity of the SK channel gating, acting as the binding site for the flexible Ca^2+^-bound CaM N-lobe. This would lead to a displacement of the S4–S5 linker away from the pore axis, subsequently expanding the S6 helices bundle and opening the cytoplasmic channel gate [55]. Since this mechanism is independent of the general swapping organization of the transmembranal core, it provides a lucid explanation of how conformational changes in the cytoplasmic domains can be allosterically coupled to channel opening in this ligand-gated type of channel.

Slo2.2: A second example of a voltage-independent S4-pore-loop/6TM1P channel in the KCa family [111], arranged in a non-domain-swapped way, is the Slo2.2 Na^+^-activated K^+^ channel [56,115]. Apart from offering some interesting insights into the allosteric mechanisms for activation of this ligand-gated channel, the cryo-EM structural data of Slo2.2 indicated that imaging a high number of particles under several conditions (e.g., different concentrations of the Na^+^-regulatory ligand), and structurally classifying them as a function of Na^+^ concentration, it is possible to obtain a titration of conformational states and their dependence on ligand levels [115]. Indeed, the data suggest that in Slo2.2 an ensemble of Na^+^-independent closed conformations may exist, preceding a highly concerted and strongly Na^+^-dependent step that steeply switches into the open conductive conformation [8,115].

BK or Slo1: Although not included in the Kv class of voltage-dependent K^+^ channels [5,7], the case of BK (or Slo1) constitutes a paradigm of not domain-swapped S4-pore-loop/6TM1P and voltage-activated K^+^ channel. Indeed, it has been largely recognized that the gating properties of Slo1 channels are controlled through allosteric mechanisms by both membrane voltage and cytosolic Ca^2+^ concentration. It is also known that, similarly to other voltage-gated ion channels, BK is arranged in a modular design, where the transmembranal core contains the tetrameric PD domain with the K^+^ permeation pore and gate, and that this domain carries four VSD domains covalently attached to the N-termini of its monomers. An additional sensory domain (the cytosolic tail domain or CTD) formed by eight RCK (regulator of conduction of K^+^) domains forming an intracellular gating ring, is also covalently attached to the C-terminus of the PD domain, providing sensitivity to Ca^2+^ and other intracellular chemical ligands such as Mg^2+^, protons, heme, CO, ethanol, and lipid molecules (reviewed in [114,115,116,117,118,119]). It is important to note that, although this modular design may point to the existence of relatively independent conformational rearrangements in each individual module in response to the corresponding stimuli, it also enables allosteric coupling between them through extensive protein–protein interactions [78]. Thus, the concept of Slo1 channels as allosterically regulated machines, dually and synergistically activated by both intracellular Ca^2+^ increases and membrane depolarization, is well established [116,118,119]. The fact that BK channels exhibit a large single-channel conductance, allowing for very detailed single channel analysis of gating, has aided to realize that their gating kinetics is complex and better explained using allosteric models ([119], see also [114,116]).

Aside from the presence of the large cytoplasmic CTD gating ring found in Slo1 and other proteins modulated by Ca^2+^ and other biological ligands [112,116], the general topology of the BK transmembranal core (i.e., the PD–VSD assembly) fits the general pattern found in the S4-pore-loop/6TM1P Kv channels, with some subtle variations that can be related to some of the functional differences exhibited by these two channel types. Thus, unlike the prototypical four transmembrane helices (S1–S4) VSD of the Kv channels, the Slo1 VSD contains an extra transmembrane segment (S0) that directs the protein N-terminus towards the external side of the membrane [116,117]. There is little doubt that the Slo1 S0–S4 domain provides the voltage sensitivity to the channel [78,114,116,117]. Nevertheless, at the functional level, the VSD of BK channels also exhibits some differences as compared with the canonical S1–S4 VSD of the Kv channels: (i) mutations that neutralize the regularly spaced basic residues in Slo1 S4, have much less impact in the amount of gating charges (voltage-sensing charges) mobilized through the membrane. Indeed, every VSD of Slo1 carries only 0.6 voltage-sensing electronic charges (around 2.4 charges per tetrameric channel), much less that the typical 12–13 effective gating charges per Kv channel. This helps to explain why Slo1 channels exhibit shallow slopes in the gating charge–voltage (*Q*–*V*) and conductance–voltage (*G*–*V*) relationships, enabling them to operate in a wider range of membrane potentials and to respond to changes in intracellular Ca^2+^ with a large shift in the half-activation voltage [78,114,116,117]. (ii) Unlike the case of the Kv channels, Slo1 residues outside the S4 segment significantly contribute to voltage sensing. These include an acidic residue at the C-terminus of S4, two charged residues in S2 and an aspartate in S3. Thus, Slo1 voltage-sensing charges are less and decentralized as compared to the Kv channels, apparently leading to smaller movements of the VSD transmembrane helices during BK voltage sensing and gating [78,112,114,115]. (iii) Apart from its role in folding of Slo1 VSD [114,116], the additional S0 segment can be important for voltage sensing, since its extracellular end appears located in close proximity to those of S3 and S4, and mutations introduced at this portion of S0 alter the activation voltage dependence of the channel [78,114]. (iv) The recent elucidation of a full-length structure of an *Aplysia californica* Slo1 channel [53,54], demonstrates that its molecular architecture deviates from that of the classical Kv1 to Kv9 channels, resembling the non-domain-swapped structure of Kv10–12 and other voltage-dependent and -independent channels (see above). This means that in this case, due to the presence of a very short S4–S5 linker, the VSD of a Slo1 subunit interacts with the PD of the same subunit, not the adjacent subunit as in *Shaker*-like channels. 

Noticeably, unlike the non-domain-swapped arrangement of the Slo1 VSD–PD assembly, a domain-swapped organization is observed between the gating ring Ca^2+^-sensing cytoplasmic domains (CTDs) and the PD–VSD transmembranal core. As indicated below, this is reminiscent of the similar arrangement encountered in Kv10–12 channels. Importantly, the new *Aplysia* structures also show that the top of the gating ring forms an extensive protein–protein interface (585 Å^2^ per subunit in the Ca^2+^-liganded structure) with the lower regions of the VSD and the S4–S5 linker, this being modified when changing from the Ca^2+^-bound to the Ca^2+^-free conditions [53,54]. Since the CTD is also directly/covalently linked to the bottom of the pore helix S6 by the so-called C-linker, a coherent model of allosteric regulation of Slo1 has been proposed in which the Ca^2+^-induced tilting of the RCK1 N-lobes of the CTD pulls the S6 helices from their own subunits, but also indirectly regulates pore gating through its not-swapped and non-covalent interactions with the VSD and S4–S5 interface [54]. As evidenced by those authors [54], and more deeply discussed by others [78], some details of the proposal can be biased due to the fact that the *Aplysia* channel may show some unusual structural and functional characteristics when compared with mammalian Slo1 channels. Also, the question of gate location remains unresolved in the new structures ([54,78], see also [119]). To what extent the direct and the indirect mechanisms contribute to channel activation remains somehow speculative. Finally, the new structures have been compared both in Ca^2+^-bound and Ca^2+^-free conditions and they unambiguously show the linkages between the transmembranal core and the CTDs, pointing to a reciprocal relationship between the voltage- and Ca^2+^-sensing machineries. However, structural details about the specific effects of voltage on the structure and their relationship with the CTD rearrangements have not been provided. 

CNG and HCN: Cyclic nucleotide gated (CNG) and hyperpolarization-activated and cyclic nucleotide-gated (HCN) channels are the two groups that constitute the family of cyclic nucleotide-regulated channels ([120], but see also [5,79]). CNG channels are essentially nonselective cation channels that are permeable to several monovalent cations and Ca^2+^, almost insensitive to membrane voltage and directly gated by binding of cyclic nucleotides (e.g., cGMP and cAMP) to intracellular cyclic nucleotide-binding domains (CNBDs). HCN are channels weakly selective for K^+^ over Na^+^ and less permeable to Ca^2+^, that are activated by changes in membrane voltage. However, in contrast to most voltage-gated channels, they are opened upon hyperpolarization and closed at positive potentials. Furthermore, their activity is enhanced upon binding of cyclic nucleotides to their intracellular CNBDs, that accelerates the activation kinetics, shifts the activation curve to more positive voltages and increases the maximal currents [79]. The presence of an intracellular CNBD is a common structural feature shared by CNG and HCN, but also by KCNH (Kv10–12) channels, that for this reason have been sometimes grouped together in the named CNBD family [79]. Nevertheless, aside from other common structural characteristics (e.g., the presence of a non-domain-swapped architecture, see below), the voltage-dependence, selectivity, and cyclic nucleotide regulation of the KCNH channels are different from those of the CNG and HCN channels. 

Either due to its direct role activating the CNG gating or to its less direct effect as regulator of the HCN channels, it is obvious that binding of cyclic nucleotides to the CNBDs must allosterically control the operation of these channels. Again, the recent cryo-EM elucidation of the tridimensional structure of TAX4 and LilK (two CNG channels from the bacteria *Leptospira licerasiae* and *Caenorhabditis elegans*, respectively [58,59]), and that of the human HCN1 in the presence and the absence of cAMP [57], has yielded new insights about possible mechanisms involved in the activation of these entities. Previous structures of HCN C-terminal fragments without transmembranal regions indicated clear cyclic nucleotide-triggered reorganizations around the CNBD regions, but few changes were observed in the organization of the C-linker domain located between the end of S6 and the CNBD in response to cyclic nucleotide binding [57,79]. This region forms a gating ring direct and covalently linked to the base of the pore, acting as a “transduction module” between this pore and the CNBDs. Also, the C-linkers may influence the state of the VSDs through their interactions with the distal S4 and/or the S4–S5 linker [57,79]. The mentioned cryo-EM structures provide some clues to better understand the allosteric mechanisms involved in cyclic nucleotide binding-induced opening and/or modulation of activation. Thus, by lowering the strength of the interactions between the gating ring helices in the cyclic nucleotide unbound state [59] and/or due to a counterclockwise rotation of the intracellular region below S6 [57,79], an iris-like dilation of the S6 helical bundle is induced in response to cyclic nucleotide binding [121]. Although a non-domain-swapped architecture is present in the transmembranal core of these channels, the domain-swapping in the cytoplasmic regions in which the C-linker/cNBD region of one subunit is rotated and interacts with that of a neighboring subunit, could be important for this goal. Note, however, that although this bundle does dilate during CNG channel activation, it does not control ion permeation since the pore gate is located at the channel selectivity filter. Thus, it has been proposed that the conformational changes of S6 are not crucial events during gating of CNGA1 channels [122]. Therefore, it remains unclear how the reorganizations/rotation of the gating ring are propagated up to the selectivity filter gate [59,79]. It is tempting to speculate that the interactions between the C-linker and S4/S4–S5/S5 regions, and the S4, S5, and S6 helices packing inherent to the non-domain-swapped core architecture of these channels, play an important role in this propagation.

In the case of the HCN channels, on the other hand, comparison of the HCN1 structures reveals only slightly rotated cAMP-bound intracellular domains in the liganded conditions, respect to the unliganded ones [57]. Since the primary permeation gate of these channels is located at the distal intracellular end of the pore (i.e., at the S6 helical bundle) and they only became activated by membrane hyperpolarization, it is likely that the pore is held closed in the depolarized conditions at which the structures are obtained, and the interactions between the VSDs and the cAMP-bound intracellular domains prevent them from further rotation [79]. This would be consistent with: (i) the existence of a network of interactions between the upper surface of the C-linkers and the S4/S4–S5/S5 region, and (ii) the facilitation of voltage-dependent opening by cyclic nucleotides, although these are not enough by themselves for channel opening in the absence of membrane voltage changes [57].

As previously mentioned, it is important to emphasize that HCN channels show an inverted gating polarity, such that depolarization is associated to closing and it is membrane hyperpolarization which triggers channel activation. However, their general tetrameric architecture is analogous to that encountered in the rest of the S4-pore-loop/6TM1P channels [57]. Indeed, the S4 movement in response to changes in membrane voltage is the same as in other voltage-dependent channels, going outwards upon depolarization and translating inwards with repolarization [123,124]. Therefore, it is the different VSD to PD coupling, and not an opposite voltage-dependent reorganization of the VSD, which causes the inverse polarity of gating exhibited by the HCN channels. According to the last hHCN1 cryo-EM structural data, a unique feature of the hHCN1 VSD is the presence of a very long S4 helix, containing two additional helical turns that prolongs its intracellular end further away from the cytoplasmic membrane surface [57]. This brings the C-terminal end of S4 and the S4–S5 linker into contact with the C-linker of a neighboring subunit, allowing the gating ring located next to the S6 helical bundle to stabilize the closed state of the cytoplasmic gate when the VSD is in the depolarized/activated conformation. This could be also favored by the presence of an exclusive cytoplasmic HCN domain and by the S4, S5, and S6 helices tight packing. Altogether this is also coherent with the not domain-swapped organization of the HCN transmembranal core and the very short and not α-helical S4–S5 linker between the VSD and the PD domains [57]. Two interesting features of this hypothesis are: (i) the depolarized conformation of the VSD would stabilize the closed state of the pore, that would spontaneously move to an intrinsically preferred open state upon membrane hyperpolarization ([28,57,79]; see also below); and (ii) as indicated for the CNG channels (see also below), interactions between the S4/S4–S5/S5 region and the cytoplasmic C-linker (and HCN) domains could also be crucial for allosterically communicate the VSD conformational changes to inverse gating in HCN channels [57]. This is consistent with previous functional studies ([125,126,127]; but see also [28,128]). Additionally, mutagenesis and subsequent functional analysis suggest that the N-terminus of HCN4 (a highly flexible region that is not resolved in the presently available HCN structures) can be important for channel activation [129]. On the other hand, the two features could explain recent data with S4-S5 split channels showing that (as previously shown with Kv10.1 and Kv11.1 channels [25,26,27], see below), covalent linkage between the VSD and the PD is not required for hyperpolarization-dependent activation or ligand-dependent gating of sea urchin spHCN channels [28]. Interestingly, as in the case of Kv10.1 [25,26] (but see [27]), in the split spHCN constructs neither the physical continuity or the presence of the linker itself, are necessary for VSD–PD coupling, that otherwise requires the S4 C-terminal and the S5 N-terminal regions. Furthermore, hyperpolarization-induced opening of spHCN is observed despite the fact that the S4 helix is not as long as that encountered in the hHCN channels [28]. Unfortunately, the cAMP-bound and -unbound cryo-EM hHCN structures have been obtained under depolarizing conditions (at zero potential) [57], and a hyperpolarized-state HCN structure is not available. Therefore, some details about how inward movement of S4 could lead to HCN pore opening still remain somehow speculative [79].

### 5.2. Evidence of Long-Range Allosteric Influences in VSD–PD Coupling of Non-Swapped Kv Channels

Participation of allosteric mechanisms on gating is not restricted to ligand-operated channels, as exemplified by BK/Slo1 with their dual ligand-voltage regulation of gating, and by the HCN channels with their inverted gating polarity and cyclic nucleotide-dependent activation. The possibility that these mechanisms can influence gating in some genuinely voltage-dependent channels of the Kv class, such as Kv10.1 (eag1) and Kv11.1 (hERG), in which a non-swapped domain organization has been recognized [51,52], is considered next. Noticeably, in these cases, a crucial role for different cytoplasmic domains acting either as essential components of the gating machinery itself, or as important regulators of the gating process, has been proposed.

In a pioneer study with Kv10.1 (eag1) and Kv11.1 (hERG) channels in which the covalent link between the VSD and the PD was broken at the level of the S4–S5 linker (S4–S5 split channels), an almost unperturbed voltage-dependent activation gating was observed [25]. This result indicated that these channels cannot use for voltage-dependent activation a classical electromechanical lever system, in which movements of the VSD are transduced to channel opening via covalent linkage of S4 to the PD by a long α-helical S4–S5 linker that, acting as a mechanical lever, pulls the gate through a noncovalent interaction with S6 [31,44,74,76,130]. The subsequent recognition of the non-domain-swapped architecture in eag1 [51] and hERG [52], in which the S4–S5 linker is very short, further supported this view. Therefore, the question arises: what other molecular mechanism(s) and/or specific variations could be involved in voltage-dependent activation of these entities?

Comparison of eag1 and hERG structures might provide some insights about this issue. Thus, although both structures were determined at a nominal voltage of 0 mV at which the VSDs are in the depolarized/activated conformation (indeed, the observed structure of the VSD domains are almost identical in both cases), the hERG intracellular gate at the S6 helical bundle is open, whereas the cytoplasmic gate of eag1 remains closed due to the inhibitory effect of the Ca^2+^/CaM complex bound to the cytoplasmic face of the channel [51,52]. Providing that the Ca^2+^/CaM-induced closed conformation of the eag1 pore is representative of the similar VSD-dependent closed conformation of both channels, the structures may suggest that opening of the intracellular gate involves a kink of the S6 helix at a glycine gating hinge located below the selectivity filter, at residue G648 in hERG and G460 in eag1 [52,70]. This would be consistent with the fact that S5 and S6 helices establish extensive anti-parallel contacts between them, whereas the intracellular end of S4 interacts with S5 and/or the intracellular portion of S6 and the C-linker [51,52]. It also would fit with the proposal that, in these cases, the inward and centric displacement of S4 would close the S6 helical gate, allowing the VSDs to compress the S5 helices and transmit force through the S5–S6 interface to the C-linker/intracellular end of S6, without needing an intact S4–S5 linker for VSD–PD coupling [51,52]. Nevertheless, at least in the case of hERG, the S6 glycine kink is contradictory with previous data showing that S6 glycines are only required for the tight packing of the channel helices, but not as gating hinges for voltage-dependent activation gating, because the S6 helices of hERG are inherently flexible [131]. Therefore, further functional and structural data with the VSD in both depolarized and hyperpolarized conformations would be necessary to ascertain if the mentioned eag1 closed state corresponds to that achieved through voltage sensor reorganizations triggered by changes in membrane voltage.

**Figure 3:**
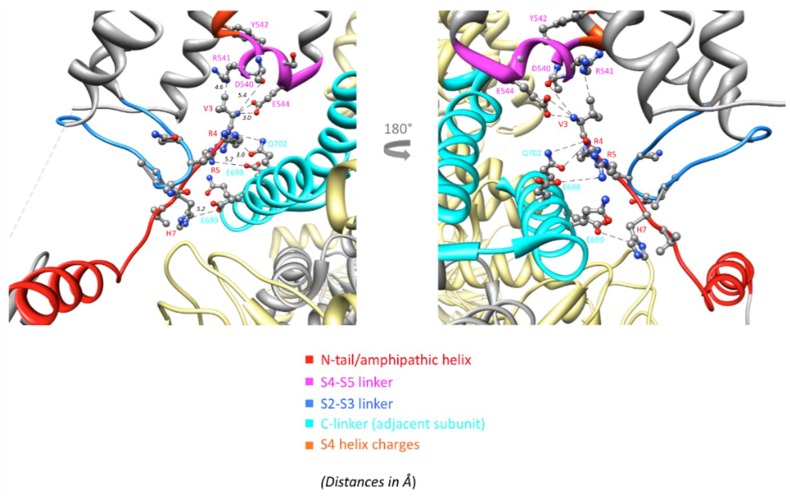
Network of interactions of the Kv11.1 (PDB: 5VA2) N-tail with the S4–S5, S2–S3, and C-linkers of the channel. Enhanced views of the region below the S4–S5 linker are shown with ribbons corresponding to the indicated domains and residue symbols colored as indicated at the bottom. Dashed lines indicate interatomic distances ranging between 3.0 and 5.5 Å. Reproduced from [27].

**Figure 4:**
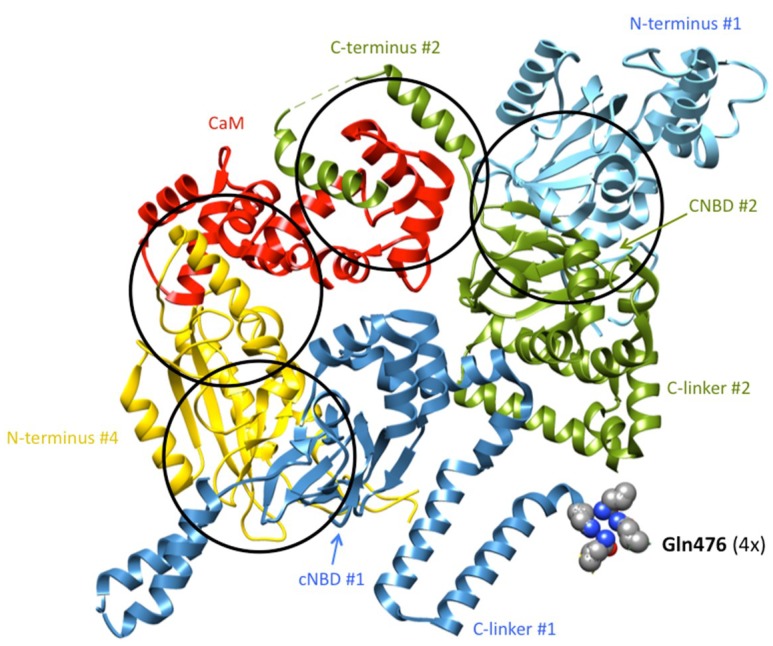
Intricate arrangement of cytoplasmic domains and CaM in Kv10.1 (eag1) channels (PDB: 5K7L). Three out of the four subunits of the channel establish contacts. The C-terminus of subunit #1 (blue) interacts with the N-terminus of subunit #4 (yelow). CaM (red) interacts with the N-terminus of subunit #4, and with the C-terminus of subunit #2 (green). The C-terminus of this subunit is also in close proximity to the N-terminus of subunit #1, resulting in bridging three out of the four subunits. This complex network of interactions is repeated four-fold in the full channel. The transmembrane segments and N- or C-terminal domains not implicated in these interactions have been removed for clarity. Atoms corresponding to residues Q476 at which the cytoplasmic gate is located are shown as spheres.

An important breakthrough provided by the new structures is the demonstration that: (i) the initial hERG N-tail is positioned in close contact with the S4–S5 and the C-linkers (that are directly attached to the carboxy terminus of the S4 and S6 helices, respectively) and with the long S2–S3 linker (a conserved feature of the KCNH channel family) [27,52,132] (Figure 3), and (ii) although non-domain swapping exists in the VSD region of eag1 and hERG, an extensive domain swapping exists within the cytoplasmic regions, and this is increased in the case of eag1 trough interaction with CaM, which in conjunction with domain swapping between N- and C-termini establishes a bridge, encompassing three subunits (Figure 4). Although the relative relevance of the different contact interfaces remains to be established, it has been proposed that in the case of eag1, CaM acts as a molecular clamp to pull the two domains together, by binding to the cNBD and PAS from neighboring subunits. Such a clamping mechanism would translate the cNBD interacting with the CaM toward the neighboring PAS domain. Because the cNBD is connected to S6 via the C-linker, the movement of the cNBD toward the PAS domain would cause a rotation of both the C-linker and S6 to induce a 55° bend in a direction that tightens the helical bundle that forms the intracellular gate, and to close the pore [51]. Note, however, that a similar CaM-dependent regulation is not present in hERG channels. 

**Figure 5:**
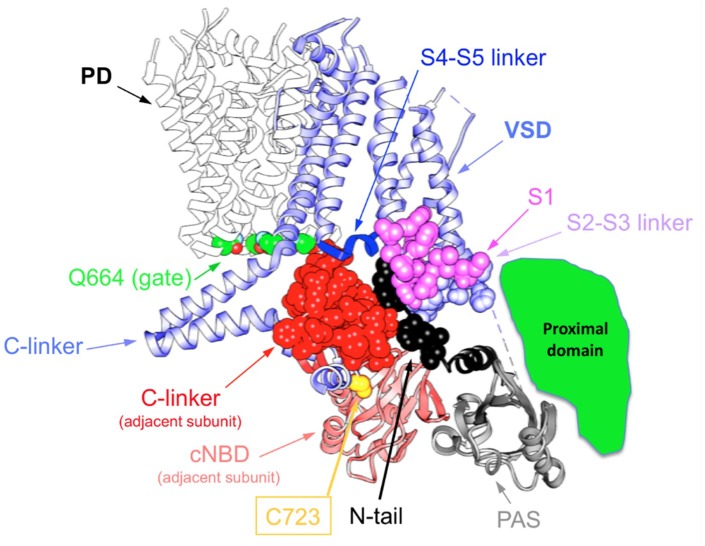
Extensive network of interactions between Kv11.1 (hERG; PDB: 5VA2) cytoplasmic domains involving several N- and C-terminal regions, plus the S4–S5, S2–S3 and C-linkers, that may dynamically contribute to modulate channel gating. A single VSD is only shown attached to the tetramer of the PD for clarity. The four residues Q664 that mark the place of the cytoplasmic gate at the S6 helix bundle are signaled. Atoms corresponding to the amino terminal N-tail (black), the S2–S3 linker (blue) and the S1 C-terminus of the same subunit (magenta), and the C-linker of the adjacent subunit (red), are shown as spheres. The positions of the PAS domain (grey), the S4–S5 linker (dark blue) and the C-linker of the same subunit, but also the cNBD of the adjacent subunit, are also signaled. Note the position of endogenous cysteine 723, known to establish disulfide bridges with cysteines engineered at the initial residues of the N-tail under different conditions [140,141]. The lower right green area indicates the approximate and speculative location of the proximal domain, that was mostly deleted from the constructs used to obtain the cryo-EM hERG structures.

Nevertheless, based in previous functional data and partial structures of some cytoplasmic regions, a role for interactions between different soluble cytoplasmic domains and the transmembrane channel core on KCNH channels gating characteristics has repeatedly been proposed. These included contacts between the N-terminal tail and the S4–S5 and C-linkers, and between the eag/PAS and cNBD domains (reviewed in [4,79,133,134,135,136,137,138,139]). Indeed, we proposed that a dynamic network of interactions involving the N-terminal tail, the S4–S5 linker and the C-terminal portion of S6, but also other cytoplasmic regions such as the eag/PAS domain and the C-linker/cNBD regions, constitutes either an essential component of the hERG gating machinery or an important regulator of the gating process [27,140,141,142,143,144,145] (Figure 5). Due to the very similar structural arrangement of these regions in the case of eag1, it has been also proposed that the connection of each of four cNBDs with the C-linker of the same subunit provides a conduit for the effect of the cNBD on the pore gate via its backbone linkage with the C-linker [146]. At the same time, the interaction of the cNBD with the PAS domain of the neighboring subunit and that of the PAS domain with the S4–S5 linker and the VSD via the N-tail, would provide a second way to control the gate and the VSD [146]. Alterations in the S4–S5 linker itself, or in any of the components involved in this dual pathway connecting the gating assembly located below the VSD/S4–S5 linker/gate interface with the gating components, had been also recently viewed as candidates to explain the impairment of a putative allosteric gating when the covalent link between VSD and PD is broken in some hERG channels split at the S4–S5 linker [27]. Thus, based in recent studies in which the gating characteristics of eag1 and hERG channels with the split point moved along the S4–S5 linker are compared [26,27], we elaborated an integrative model of hERG gating. Accordingly, by actively pushing the C-terminal end of the S4 helix and/or the initial section of the S4–S5 linker against the S5–S6 PD module, the closing of an otherwise intrinsically stable open pore can be favored at negative potentials.

Covalent breaks at the N-terminus of the linker can also influence S4 helix mobility and the general structural reorganizations of the VSD, due to the role of S4–S5 as integrator of signals coming from cytoplasmic regions, such as the N-terminal PAS and the C-terminal cNBD domains and C-linkers [27]. Interestingly, the eag1 and hERG results with split channels lead to the proposal that the isolated PD may prefer either a basal closed [26] or an intrinsically open state [27]. However, in both cases the C-terminal region of helix S4 has been considered important for the entry into a stable closed state, suggesting that the VSD acting as an inhibitory module is necessary to maintain the channel closed at negative potentials [26,27,79]. Although expression of an isolated PD yields no detectable currents, arguing in favor of a stable closed conformation under these conditions, it is possible that the energetic profile of this isolated module is different from that of the complete VSD/PD assembly, that may favor a preferentially opened basal conformation [27,147]. Finally, through a combination of mutagenesis, electrophysiology, and structural modeling using eag1 as a representative template for the hERG closed pore, a similar interplay between N-tail, eag/PAS and VSD domains, S4-S5 and C-linkers, and cNBD has been recently envisioned as an important contributor to hERG gating kinetics [133]. This further supports the view that interactions between soluble domains and the TM part of these channels are critical determinants of gating characteristics [4,27,79,132,133,134,135,136,137,138,139,140,141,142,143,144,145]. In this context, the presence of unaltered cNBD and eag/PAS domains could be crucial for appropriately position the N-terminal tail, to interact with the gating machinery. Interestingly, the constitutive activity exhibited by eag1 channels covalently interrupted within the C-terminal S4 can be reverted to a wild type-like closure by point mutations in the first residue (D342) of the C-terminal demi-channel or by a structural alteration at the amino terminus of the N-terminal demi-channel [26]. Nevertheless, the interaction partner of D342 and the reason for the similar effect of the amino terminal alteration remain to be established. Noticeably, the absence of the N-tail region not only causes effects on gating similar to those induced by removal of the whole eag domain [143,148,149,150,151,152], but also determines the loss of mode-shift (also named voltage-dependent potentiation) regulation caused by rearrangement of the cNBD intrinsic ligand and/or neighboring regions in zELK KCNH channels [153]. Also, the close proximity of the N-tail to the intracellular S2–S3 linker (Figure 3 and Figure 5) and the alterations of hERG gating caused by disruption of this linker, similar to those triggered by covalent breaks of the VSD S4 at its C-terminal end, suggests that S2–S3 may be an important part of the gating machinery [144]. Further work would be necessary to know in a more direct way what kinetic and conformational step is determined by every specific interaction and how they may contribute to the gating mechanism. 

## 6. Concluding Remarks

The combination of functional and structural evidences points to gating of KCNH channels, but also of other members of the Kv family, as a dynamic and multi-domain question. The relevance of the S4–S5 linker for transferring as a lever the movement of S4 into opening the gate is clear for Kv1 to Kv9 channels, but other mechanisms are required to explain the behavior of other channels. We refer to such complementary mechanisms as ‘allosteric’ to highlight the importance of cytoplasmic domains, relatively distant and not continuous with the gate-forming loops, and whose relevance is demonstrated with structural and functional evidences. The intimate molecular mechanisms underlying such allosteric gating are however not yet clear. Therefore, in this case the static view provided by the structures should be complemented by more dynamic information based in mutagenesis, functional and/or fluorometric assays, kinetic modelling and in silico molecular dynamics simulations. Nevertheless, the possibility of using cryo-EM as a tool to capture different conformational states of the same protein, in proportion to their steady-state distribution [8], or in the presence of different pharmacological probes [88], increases the interest on this methodology. This could be reminiscent of the great contribution to understand the working mechanism of P-type ATPases, provided by elucidation of the crystal structures of Ca^2+^-ATPase in different conformational states [154,155,156,157,158,159]. Some remarkable steps in this direction are: (i) the recent comparison of SK channel activated and deactivated states, through three-dimensional classification of cryo-EM particles, to gain insights about the structural basis for channel activation [55], and (ii) the structural titration of the Na^+^-activated K^+^ channel Slo2.2 with increasing concentrations of Na^+^, to detect an ensemble of closed conformations that do not depend on Na^+^ concentration, followed by the emergence of an open conformation in a highly Na^+^-dependent way, without evidence of Na^+^-dependent intermediates [113,160]. Although in these cases ligand-dependent channels are used for these studies, it is conceivable that in a near future, voltage-gated channels trapped in a specific functional state (e.g., with an activated or deactivated VSD) can be used for high resolution ‘random spherically constrained’ (RSC) single-particle cryo-EM structural analysis, following their reconstitution in spherical lipid vesicles (liposomes) to which the desired transmembrane potential is imposed [161,162,163]. Noticeably, the view of voltage-dependent channel gating as a multi-domain question, also involving several cytoplasmic regions, is not exclusive of KCNH and other Kv channels, but also encompasses other entities such as Cav and Nav channels [2,47,48,49,164,165,166]. Therefore, fun seems to be assured for the next years in the gating world of the Kv channels and their relatives.
